# Safety of tofacitinib in IBD: A tricky puzzle

**DOI:** 10.1002/ueg2.12587

**Published:** 2024-05-18

**Authors:** Laurent Beaugerie

**Affiliations:** ^1^ Department of Gastroenterology Sorbonne Université INSERM Institut Pierre Louis d’Epidémiologie et de Santé Publique AP‐HP, Hôpital Saint‐Antoine Paris France

**Keywords:** cancer, cardiovascular risk, Crohn's disease, inflammatory bowel disease, safety, serious infections, tofacitinib, ulcerative colitis

In this issue of the United European Gastroenterology Journal, Panes et al. report the updated safety data of the development program of tofacinitib, the first JAK inhibitor authorized in ulcerative colitis.^1^ They conclude that herpes zoster and serious infections (SI) are the only events whose frequency exceeds the symbolic threshold of 1 per 100 patient‐years (meaning a 10% risk for a 10‐year exposure), and that safety profile does not change significantly with prolonged exposure (up to 9 years).

Fifty years have been necessary to demonstrate that azathioprine promotes lymphomas in IBD.^2^ Fortunately, establishing the safety profile of new molecules is easier and faster, thanks to adequately powered cohorts and medico‐administrative databases. In the case of tofacitinib, a major breakthrough was additionally made by a dedicated huge randomized safety trial,^3^ even if eligible patients had a particular profile (rheumatoid arthritis patients over the age of 49 and with cardiovascular risks).

As for any molecule, the safety profile of tofacitinib can be categorized into four chapters: molecule‐specific biological/organ toxicity, promotion of cardiovascular events, SI and malignancy.

Tofacitinib‐induced biological abnormalities (neutropenia, lymphopenia, changes in lipid profile) were detected in the development program in rheumatology. This safety point is now well defined, provided that clinicians respect recommendations of the summary of product characteristics. The excess risk of major arterial events and venous thromboembolism events was also quickly identified and gave rise to evolving warnings and restrictions of indications issued by drug agencies. It is now established that tofacitinib promotes cardiovascular events in rheumatoid patients over the age of 49 with at least one cardiovascular risk factor,^3^ even if we cannot exclude that adalimumab, the comparator, could have a protective effect against cardiovascular events, as suggested in IBD,^4^, ^5^ which could artificially penalize tofacitinib. The extent of the excess risk is probably much lower, or even non‐existent, in young and middle‐aged patients with no significant cardiovascular risk in rheumatoid arthritis.^6^ The dose‐dependent excess risk of zoster related to the exposure to tofacitinib^7^ is established. In older patients receiving tofacitinib 10 mg twice daily, the level of the risk almost reaches 100% for a 10‐year exposure^8^! However, the availability of recombinant vaccines has radically changed the landscape,^9^ paving the way for preventing all forms of zoster in the majority of patients.

The two important missing pieces of the puzzle in IBD are the excess risk level of SI (excluding zoster) and malignancy. The excess risk of SI related to a new molecule cannot be established in placebo‐controlled randomized controlled trials. Indeed, uncontrolled inflammation in the placebo arm promotes all forms of infections,^10^ including viral^11^ and extra‐intestinal ones,^10^, ^11^ which biases the comparison. Here again, the results of the New England trial are determinant.^3^ There was a significant 48% excess risk of SI in patients receiving tofacitinib 10 mg daily (no significant risk for 5 mg daily), keeping in mind that patients exposed to antiTNF monotherapy have been show in IBD to have a two‐fold increased risk of SI compared to patients receiving no immunosuppressant.^12^ In addition, the risk of death by SI must be considered. In the French national database, the mortality rate in patients developing SI was 1% up to the age of 65 years, and 10% after.^12^ Clearly, the risk of SI must be taken into account when initiating tofacitinib in frail/aged patients.

The risk of drug‐induced cancer is the dread of clinicians. Data from development programs are not useful in this matter, because of a lack of power and the absence of relevant control population. In rheumatoid patients over the age of 49, there was a 48% excess risk of cancer in patients exposed to tofacitinib versus those exposed to adalimumab. Adalimumab can be considered here as a neutral comparator since no study has demonstrated an overall excess risk of cancer associated with anti‐TNF agents.

Whether the excess risk of cancer in rheumatoid patients can be transposed to IBD patients of similar age is plausible, but uncertain. Different immune statuses according to distinct diseases may impact or not on the risk level of drug complications. For instance, the incidence of azathioprine‐induced pancreatitis is much higher in patients with IBD than in patients with autoimmune hepatitis.^13^ By contrast, the risk of EBV‐related lymphoma has a similar level in patients with IBD and in transplanted patients.

Years of patience will be necessary before knowing if tofacitinib and other JAK inhibitors increase the risk of cancer in IBD, and in particular the risk of EBV‐related lymphoma, since JAK inhibitors significantly promote viral infections, like thiopurines do.

## CONFLICT OF INTEREST STATEMENT

The author has no conflict of interest to disclose in 2023 and 2024.
